# Within-Person Variation in Serum Thyrotropin Concentrations: Main Sources, Potential Underlying Biological Mechanisms, and Clinical Implications

**DOI:** 10.3389/fendo.2021.619568

**Published:** 2021-02-24

**Authors:** Evie van der Spoel, Ferdinand Roelfsema, Diana van Heemst

**Affiliations:** ^1^Section Gerontology and Geriatrics, Department of Internal Medicine, Leiden University Medical Center, Leiden, Netherlands; ^2^Section Endocrinology, Department of Internal Medicine, Leiden University Medical Center, Leiden, Netherlands

**Keywords:** thyrotropin, thyroid-stimulating hormone, biological variation, circadian rhythm, ageing, seasonality, within-person variation

## Abstract

**Background:**

Individuals exhibit fluctuations in the concentration of serum thyroid-stimulating hormone (TSH) over time. The scale of these variations ranges from minutes to hours, and from months to years. The main factors contributing to the observed within-person fluctuations in serum TSH comprise pulsatile secretion, circadian rhythm, seasonality, and ageing. In clinical practice and clinical research however, such within-person biological variation in serum TSH concentrations is often not considered. The aim of this review is to present an overview of the main sources of within-person variation in TSH levels, as well as the potential underlying biological mechanisms, and the clinical implications.

**Summary:**

In euthyroid individuals, the circadian rhythm, with a nocturnal surge around 02:00–04:00 h and a nadir during daytime has the greatest impact on variations in serum TSH concentrations. Another source of within-person variation in TSH levels is seasonality, with generally higher levels during the cold winter months. Since TSH is secreted in a pulsatile manner, TSH levels also fluctuate over minutes. Furthermore, elevated TSH levels have been observed with ageing. Other factors that affect TSH levels include thyroid peroxidase (TPO)-antibody positivity, BMI, obesity, smoking, critical illness, and many xenobiotics, including environmental pollutants and drugs. Potential underlying biological mechanisms of within-person variation in TSH levels can be safely concluded from the ability of TSH to respond quickly to changes in cues from the internal or external environment in order to maintain homeostasis. Such cues include the biological clock, environmental temperature, and length of day. The observed increase in TSH level with ageing can be explained at a population level and at an organism level. In clinical practice, the season for thyroid testing can influence a patient’s test result and it occurs frequently that subclinical hypothyroid patients normalize to euthyroid levels over time without intervention.

**Conclusions:**

Serum TSH concentrations vary over time within an individual, which is caused by multiple different internal and external factors. It is important to take the within-person variations in serum TSH concentrations into account when testing a patient in clinical practice, but also in performing clinical research.

## Introduction

Hormones of the hypothalamus-pituitary-thyroid (HPT) axis are part of a feedforward and feedback system. Thyroid stimulating hormone (TSH) is a key player in this system, which is designed to respond quickly to changes in the environment aiming to maintain homeostasis in the human body. By adapting the level of TSH, circulating levels of thyroxine (T4) and partly triiodothyronine (T3) are controlled and kept within a normal range (1, 2). Indeed, it is known that TSH levels can vary over time within a person.

Among other, TSH levels are influenced by drugs, acute and chronic illness, undernutrition, the biological clock, seasonality, pregnancy, and by other hormones, e.g., cortisol. However, the magnitude and the importance of variation in TSH levels within a person over time are not completely clear yet. Consequently, the influence of variation in TSH levels on diagnosis and study outcomes might be underestimated. In this review, we discuss the magnitude of the variation in TSH levels and the potential sources (internal and external) that may contribute.

Variation in TSH levels can be caused by biological and non-biological variation, the latter includes pre-analytical and analytical variations, and has been reviewed by others (3). In the current review we focus on biological variation, in particular on within-person biological variation. Within-person variation in TSH levels is caused by rhythms ranging from minutes to years, including pulsatile secretion, circadian rhythm, monthly changes, and seasonality (3, 4). Furthermore, TSH levels change with age, with in general higher levels with increasing age (5). Independent of time, within-person variation in TSH is also caused by effects of among others medication, illness, TPO antibody positivity, and iodine intake.

Medical practice and guidelines have been traditionally based on the “average” patient and how the “average” patient responds to various treatments, as informed by randomized clinical trials. Especially vulnerable patients, which include older patients with comorbidities and polypharmacy, are underrepresented in clinical trials. Consequently, evidence on how such vulnerable patients respond to treatment is lacking (6). As it is becoming increasingly clear that patients are a heterogeneous group, in current practice and guidelines, there is increasing recognition of the need to individualize management of a patient’s disease. As a promising strategy toward personalized medicine, N-of-1 studies have been proposed as a design to determine the best treatment for individual patients (7). In a N-of-1 trial, each individual receives several treatments in a multiple crossover design, allowing determination of the best intervention for an individual patient. However, a drawback of this type of study is that different points in time are compared with one another, and thus results may be influenced by fluctuations that occur within an individual over time (8).

In this context, a lack of awareness among clinicians of variation in TSH levels within individual patients over time could lead to under- or overdiagnosis of (subclinical) thyroid diseases, or to inadequate prescription of thyroid medication to patients with thyroid disease. Furthermore, if the variation in TSH levels is not considered, this could lead to a distorted interpretation of outcomes and results in scientific research. Therefore, in this review we give an overview of the main sources of within-person variation in euthyroid individuals to provide insight into TSH variation within the normal range. Additionally, available research on TSH variation in patients with thyroid disease and/or using thyroid medication will be discussed. Moreover, the potential underlying biological mechanisms and the clinical implications of within-person variation in TSH levels are reviewed. Much is known about the short-term within-person variation in TSH levels. However, long-term variations in TSH levels are rarely investigated in longitudinal studies and more often in cross-sectional studies. In this review, evidence from both cross-sectional and longitudinal studies is described. Literature research was mainly performed using PubMed in the first 8 months of 2020.

## Sources of Within-Person Variation in TSH Levels

### Pulsatile Secretion

TSH is secreted in a basal (non-pulsatile) and in a pulsatile manner by the anterior pituitary gland (9, 10). The feedback control by mainly T3 and T4 from the thyroid gland, together with the feedforward control by mainly thyrotropin-releasing hormone (TRH) from the hypothalamus, tightly regulate TSH secretion. It is also important to note that other hormones, including leptin, somatostatin, and dopamine, are known to influence the secretion of TSH, albeit to a lesser extent (11–15). The frequency, size, and duration of a secretory burst (i.e., pulse) of TSH is determined by the interplay between these feedback and feedforward signals.

#### Studies on Pulsatile Secretion

In 1978, Weeke et al. found short-term variations with a mean cycle-length of 31 min in serum TSH concentrations measured in blood withdrawn every 5 min starting in the evening for 6 to 7 h in five healthy men (4). Using spectral analysis together with periodic regression analysis in a study with frequent blood sampling, pulsatile secretion of TSH was detected with periodicities of 60 or 85–100 min in six out of the ten healthy volunteers (10). The mean pulse frequency of TSH levels was found to be 13 pulses (range 10–18) per 24 h in blood withdrawn every 10 min in six healthy subjects (9).

#### Deconvolution Analysis to Detect Pulsatile Secretion

Several techniques can be used to determine pulsatility in TSH serum concentrations, but deconvolution analysis is most commonly used in 24-h time series data. Keenan et al. developed a deconvolution analysis algorithm by which a 24-h hormone concentration profile is decomposed into underlying secretory bursts, basal secretion, elimination of previously secreted hormone, and random experimental variability (16–18). Therefore, deconvolution analysis can be used to assess the frequency, size, and duration of a hormone pulse. The algorithm in the software program MATLAB (the MathWorks, Inc., Natick, MA) first detrends the data and normalizes concentrations by converting them to values between 0 and 1 (18). This step is performed to normalize the span of diverse values in separate datasets so that all have a comparable chance of detecting pulses, despite variable baselines. Second, successive potential pulse-time sets, each containing one fewer burst, are created by a smoothing process. Finally, a maximum-likelihood expectation deconvolution method estimated all secretion and elimination rates simultaneously for each candidate pulse-time set. Deconvolution analysis determines secretory bursts and thereby calculates which part of TSH was secreted in a pulsatile manner within a 24 h time period. The “remaining part” of the 24-h TSH concentration that cannot be explained by secretory bursts, is called non-pulsatile or basal. It is therefore assumed in this model that the basal secretion is constant during the day. However, it is not known whether there is a constant tonic release of TSH from the pituitary or that it might be variable over time like insulin, which was studied by using portal vein sampling (19).

#### Example of Concentration Profile and Secretion Rate

**Figure 1:**
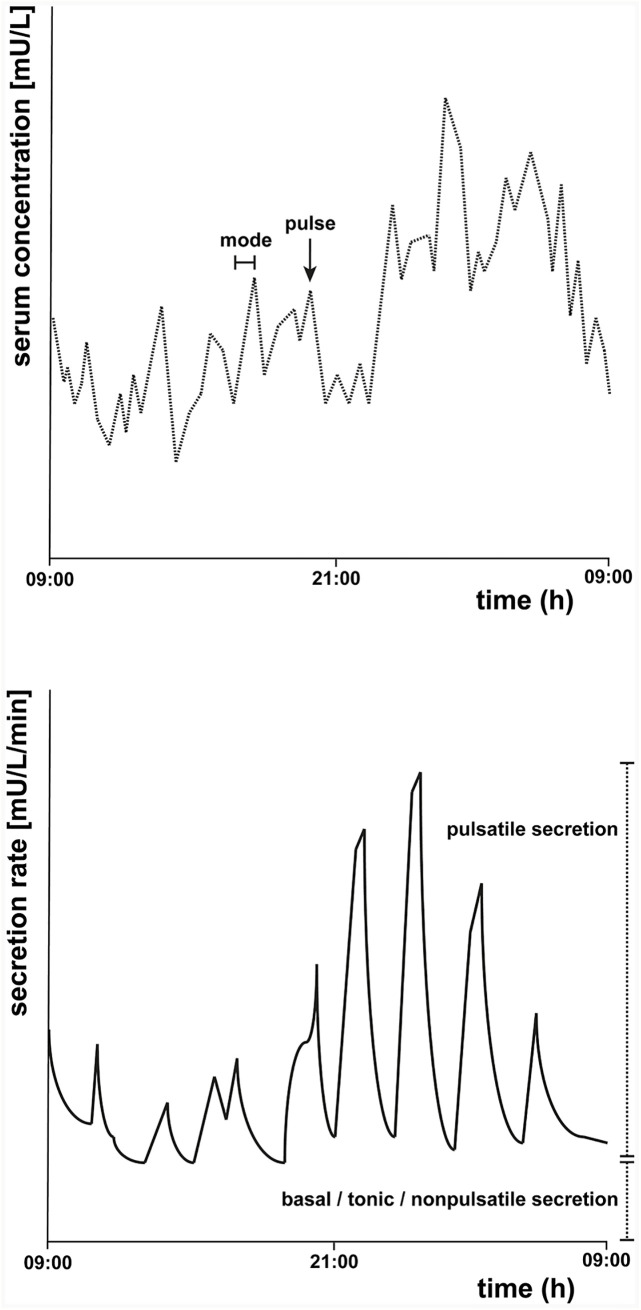
Schematic representation of a fictional 24-h thyroid-stimulating hormone (TSH) concentration profile (top) and secretion rate (bottom). The top figure represents an example of a 24-h TSH concentration profile starting at 09:00 h. A pulse and the mode of one pulse, which is the time it takes before the pulse reaches its maximum, are indicated. The bottom figure is a schematic representation of an example of the secretion rate, which can be determined by deconvolution analysis. The basal tonic (or non-pulsatile) secretion and the pulsatile secretion together contribute to the total TSH secretion.

**Figure 1** presents an example of a fictional 24-h TSH concentration profile and a corresponding fictional secretion rate diagram. In the top figure, a pulse, and the mode, which is the time it takes before the pulse reaches its maximum, is indicated. In the bottom figure, the secretion rate is depicted, with an indication of the pulsatile and the non-pulsatile part of the TSH secretion.

#### Characteristics of Pulsatile Secretion

In a study with 38 healthy individuals, including 20 women and 18 men, with a mean age of 41 years (range, 25–64 years), the mean [standard error of the mean (SEM)], number of pulses (secretory-burst frequency) was determined as 16.7 (0.091) per 24 h with a mean mass per pulse of 0.90 (0.06) mU/L (20). Furthermore, the mean (SEM) time before the pulse reaches its maximum (mode) was calculated as 20.0 (2.0) min. The fast component of the half-life of TSH, which is effected by the advection and diffusion of TSH, is generally set to be estimated by deconvolution analysis between 17 to 26 min (21). The slow half-life, which is affected by the irreversible metabolism and elimination of TSH, is set between 66 to 93 min. The mean (SEM) fast half-life was calculated as 22.6 (1.0) min and the slow-phase half-life 104 (2.9) min in these healthy individuals (20). No differences were found between men and women (22, 23). Similar results were obtained in a group of 38 healthy older individuals, including 20 men and 18 women, with an average age of 65 (range 52–76) years, although the slow half-life was lower in this group (on average 78.1 min) (24).

### Circadian Rhythm

The greatest impact on variations in serum TSH concentrations has been observed for the sampling time (25, 26), which is caused by the circadian rhythm of TSH. This daily cycle of 24 h is driven by the biological clock of the suprachiasmatic nucleus (SCN). In general, it has been observed that TSH levels reach a maximum between 02:00 and 04:00 h in healthy individuals (5, 20, 27–30). The minimum (i.e., the nadir) of the circadian rhythm of TSH occurs during daytime (4, 5, 27, 28, 31).

#### Cosinor Analysis to Detect Circadian Rhythmicity

**Figure 2:**
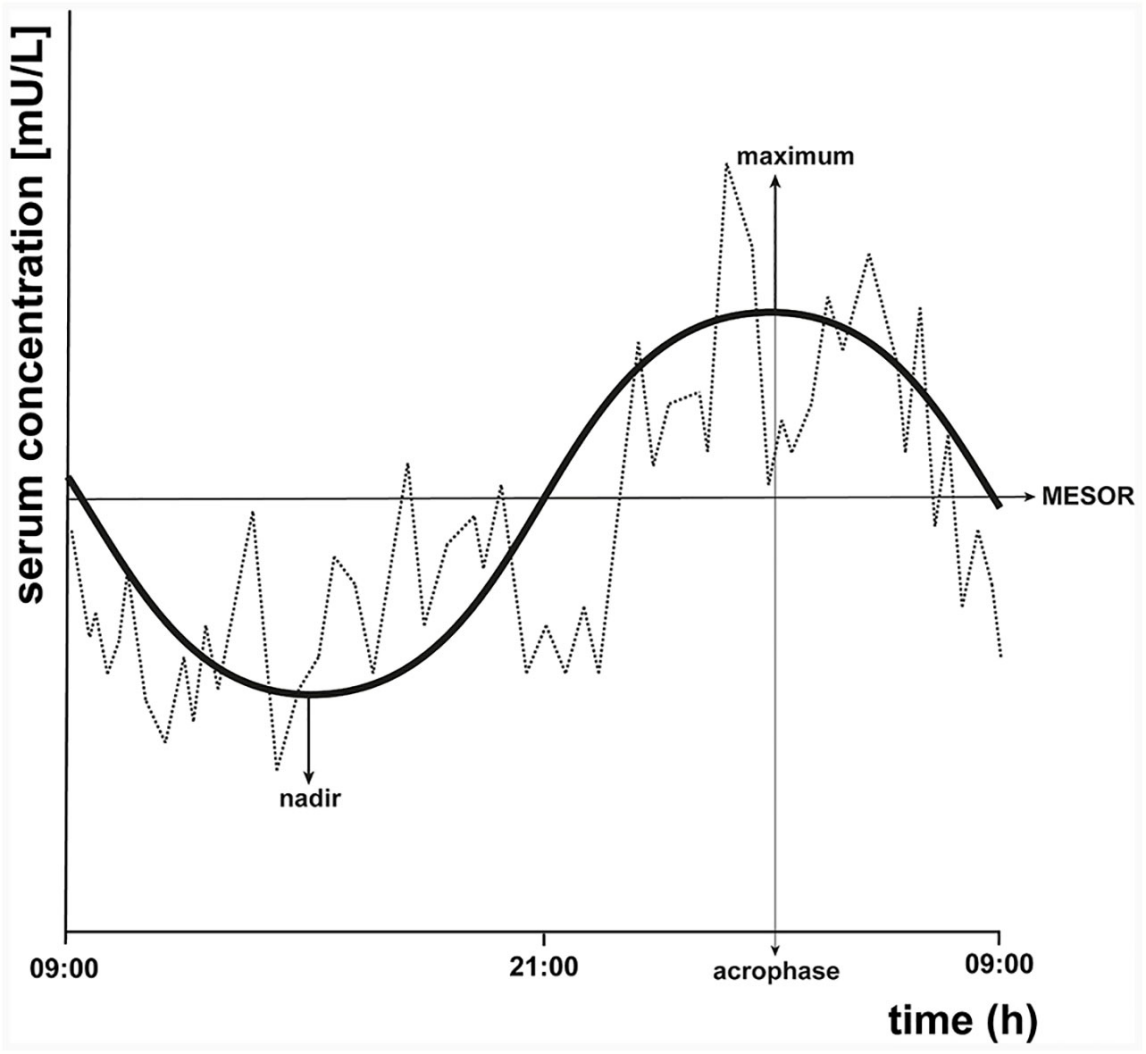
Schematic representation of a fictional 24-h thyroid-stimulating hormone (TSH) concentration profile with cosinor model parameters. A cosinor model with its parameters is drawn on the same fictional 24-h TSH concentration profile of **Figure 1** to illustrate the circadian rhythm of TSH. Cosinor analysis calculates the midline estimating statistic of rhythm (MESOR), which is a circadian rhythm-adjusted mean, the maximum, the nadir (minimum), the amplitude, and the acrophase, which is the time at which the cosinor model reaches its maximal value.

To illustrate the circadian rhythm of TSH, we included an example (see **Figure 2**) of a fictional 24-h TSH concentration profile and drawn a cosinor model and its parameters. Cosinor analysis can be used to determine whether a 24-h concentration profile displays a sinusoidal circadian rhythm. Cosinor analysis is a model-dependent method which fits a cosinor function to the raw data (32). First, the rhythm detection test, also called the zero-amplitude test, is performed to test the overall significance of the cosinor model. If this model fits significantly, it determines among others the midline estimating statistic of rhythm (MESOR), which is a circadian rhythm-adjusted mean based on the parameters of a cosine function fitted to the raw data. In addition, the amplitude is provided, which is the difference between the maximum and MESOR of the fitted curve. The acrophase represents the time of the maximal value assumed by the curve. Furthermore, the nadir, the lowest point of the cosinor model, is calculated based on the MESOR and the amplitude. Other methods that can be used to examine the circadian rhythmicity of a 24-h TSH concentration profile are for example a modified cosine function (21, 33), a locally weighted polynomial regression analysis (24, 34), or a periodic spline analysis (5).

#### Circadian Rhythm in Thyroid Disease

TSH secretion patterns have also been studied in treated and untreated primary hypothyroidism patients, which has been reviewed previously (29). In seven patients with primary hypothyroidism on T4 therapy and eight patients with primary hypothyroidism on combined therapy (T3/T4) for more than 3 months, a circadian rhythm with a nocturnal rise persisted (35). Also another study in eleven primary hypothyroid patients on stable T4 replacement therapy showed retention of the circadian rhythm in TSH secretion (36). However, in untreated primary hypothyroid patients, 24-h TSH secretion was increased and the nocturnal surge was often decreased or absent while the pulse frequency was similar compared to euthyroid controls (37–41).

#### Circadian Rhythm and Ageing

In a population of euthyroid individuals, mean levels of TSH differ with age, but some studies showed that the circadian rhythm does not change with age (5, 27). In contrast, others reported a diminished nocturnal TSH surge in older subjects compared to young controls (42, 43) and an advanced (earlier) shift of the onset of the nocturnal surge of TSH with increasing age (23).

### Seasonality

Multiple studies showed that TSH levels are subject to change of season. Evidence for such circannual variation in serum TSH comes from cross-sectional studies, but also from longitudinal studies. In general, TSH levels were highest in winter season.

#### Cross-Sectional Studies on Seasonality

Several cross-sectional studies found higher TSH levels in euthyroid individuals without thyroid disease whose blood withdrawal for TSH measurements was performed in autumn or winter seasons compared to individuals with blood withdrawals for TSH measurements in spring or summer seasons. An impressively large American study using 465,593 TSH measurements of 324,750 individuals without thyroid disease aged 1–104 years showed that the upper TSH limit of the 95% reference interval was significantly higher in December (6.06 mU/L) than in August (4.31 mU/L) for both sexes and all age groups (5). The lower limit of the 95% reference interval was 0.5 throughout the year. Barchetta et al. found in 294 Italian euthyroid subjects with a mean age (SD) of 48.5 (12.4) years higher mean TSH levels in individuals evaluated in autumn or winter seasons (2.3 ± 1.3 µU/ml) compared with individuals who had their blood samples taken in spring or summer seasons (1.8 ± 1.1 µU/ml, p = 0.03) (44). Also in a large group (*N*=206,486) of patients (health check-ups, inpatient and outpatient visits) in Peking, it was observed that median TSH concentrations were highest in winter (1.96 ± 0.128 μU/L) and lowest in summer (1.86 ± 0.111 μU/L) (45). Likewise, mean monthly TSH levels were highest in December in groups of middle-aged and older Italian euthyroid individuals using 8,310 laboratory TSH measurements (46).

#### Longitudinal Studies on Seasonality

Similar results were obtained when the seasonality of TSH levels was investigated in longitudinal studies, with repeated measurements in the same individuals. Maes et al. showed that 08:00 h fasting TSH levels were highest in December and lowest in June when performing monthly blood sampling for 1 year in 26 Belgian healthy subjects with a mean age (SD) of 38.7 (13.4) years (47). When clustered per season, highest mean (SD) TSH levels (1.85 (1.02) mU/L) were observed in autumn (21 September–20 December) and lowest (1.48 (0.72) mU/L) in spring (21 March–20 June) (47). Furthermore, TSH levels were higher in the winter and spring seasons compared to summer and fall seasons (maximal difference of 0.30 mU/L) in a large group of Korean euthyroid subjects (*N*=28,096) who had on average 3.1 tests per person during a median (interquartile range (IQR)) follow-up of 36 (22–53) months (48).

#### Studies With No Statistically Significant Seasonality

A few studies found higher TSH levels in winter/autumn compared to summer/spring, but were unable to demonstrate statistically significant seasonality in TSH. For example, no significant seasonal variation in TSH levels was found in a cross-sectional Italian study of a large cohort of 11,806 euthyroid subjects with a median (IQR) age of 49 (37–61) years (49). Nevertheless, median (IQR) TSH values were slightly higher in December [1.46 (0.90–2.20) mU/L] and January [1.46 (0.90–2.16) mU/L] compared to all other months, with lowest median (IQR) TSH levels of 1.39 (0.90–2.10) in April (49). Likewise, no significant seasonal difference was present in a small subset of 159 subjects of this study in which TSH was measured twice in the same year (49). In 152 Iraqi euthyroid subjects with a mean (SD) age of 32.24 (12.05) years who had their TSH levels measured in both summer and winter seasons, no significant difference was found between summer [mean (SD) of 2.34 (1.25) mU/L] and winter [2.25 (1.25) mU/L] measurements (50). Andersen et al. found 8% higher serum TSH concentrations in autumn and winter than in spring and summer using monthly blood samples in 15 healthy Danish men with an age range of 24–53 years, but this difference was not statistically significant (51).

#### Seasonality in Thyroid Disease

In addition, comparable annual changes were observed in Japanese treated and untreated patients with thyroid disease aged > 20 years (using 1,637,721 TSH measurements) with median TSH levels of 1.46 (range 0.01–769.1) µU/ml in winter and 1.31 (0.01–594.8) µU/ml in summer (52), and in Korean patients (mean (SD) age of 48.6 (11.6) years) with subclinical hypothyroidism without medication (*N*=1,751) with a maximal TSH difference of 0.69 mU/L (48). Median TSH levels were highest in January compared to other months in 3,934 L-T4-treated athyreotic patients with a median (IQR) age of 50 (41–59) years of an Italian cross-sectional study (49). Additionally, in a subset of 119 patients [mean (IQR) age of 46 (39–59) years] with one blood withdrawal taken in winter and one in summer, median (IQR) TSH levels were higher in winter season [0.80 (0.22–1.44) mU/L] compared to summer season [0.20 (0.06–0.70) mU/L] (49). Furthermore, although the 10 (aged 32 to 66 years) (53) and 7 (aged 27 to 66 years) (54) primary hypothyroid patients in two longitudinal Japanese studies received constant doses of levothyroxine (L-T4), their unstimulated TSH levels and TSH levels after TRH stimulation were higher in winter compared to summer. In contrast, no significant difference was found in mean (SD) TSH levels measured in 25 subclinical hypothyroid patients with a mean (SD) age of 36.44 (14.82) years in winter [6.04 (1.17) mU/L] compared to summer [6.64 (1.60) mU/L] in Iraq (50).

#### Seasonality and Ageing

Whether the circannual rhythm of TSH changes with ageing is not clear, one study showed that the seasonal changes were independent from age (55), but another study showed that the circannual rhythm of TSH was stronger in middle-aged and older subjects compared to young subjects (46). In summary, studies showed that TSH levels were higher during winter than during other seasons in both euthyroid individuals as well as in patients with thyroid disease, but the influence of age on the seasonality of TSH concentrations is unsettled.

### Ageing

Numerous studies, both cross-sectional and longitudinal, have shown that TSH levels differ with age, with in general higher mean levels with increasing age. A longitudinal study (*N*=908) with a follow-up of 13 years found that mean (SD) serum TSH concentrations increased from 1.49 (0.79) to 1.81 (0.96) mU/L with the largest difference in subjects aged 60+ years (56). Another longitudinal study in 533 healthy older individuals (65+ years) found a mean (SD) increase of 0.28 (1.4) mU/L in 13 years of follow-up (57). In a cross-sectional retrospective analysis of 465,593 TSH measurements of 324,750 individual without thyroid disease, the 97.5% reference interval increased from 6.25 mU/L in subjects aged 21–40 years, to 6.55 (41–60 years), to 6.80 (61–80 years), to 7.55 mU/L in subjects aged 80+ years (5). A similar increase was observed in another cross-sectional retrospective study in 1,388 healthy subjects with a mean (95% reference interval) of 1.30 (0.39–4.29) mU/L in the group of 20–29 years and 1.96 (0.63–6.15) mU/L in the 70+ group (58). Boucai et al. observed an increase in serum TSH concentrations over different age groups with median (2.5–97.5% interval) levels of 1.30 (0.40–3.98) in the 20-to-29-years-old-group and 1.99 (0.44–6.92) mU/L in over-80-years-old-group (59).

#### Clinical Trials in Older Persons With Subclinical Hypothyroidism

One potential school of thought states that age-related changes in hormone levels, including TSH, contribute to health problems in older individuals. It is therefore an ongoing debate whether TSH levels should be “normalized” in older individuals to prevent age-related loss in functioning or whether the upper reference limit for TSH should be adapted according to age (60). In line with the increase in mean TSH levels with age, the prevalence of subclinical hypothyroidism is also increasing, which is defined as an elevated TSH level that occurs in conjunction with a serum fT4 concentration within the normal range (61, 62). Therefore, recently, two relatively large (*N*=737 and *N*=251) randomized placebo-controlled clinical trials (RCTs) have been performed in which older persons with subclinical hypothyroidism received levothyroxine (L-T4) or placebo supplementation (63, 64). The two primary study outcomes were the change in the hypothyroid symptoms score and tiredness score from the thyroid-related quality of life patient-reported outcome (ThyPRO) questionnaire from baseline to 1-year follow-up. Although TSH levels in participants receiving L-T4 treatment declined, there was no beneficial effect on their thyroid-related symptoms compared to the placebo group (63, 64). Along the same line, a systematic review and meta-analysis provided evidence that the use of thyroid hormone therapy was not associated with improvements in general quality of life or thyroid-related symptoms in RCTs with a follow-up of 3–15 months (65).

#### TSH and Human Longevity

In fact, higher TSH levels might even be beneficial for healthy ageing and longevity. Older individuals from the Leiden 85-Plus Study with elevated serum TSH levels without thyroid disease were not at risk of increased morbidity and may have a prolonged life span (66). We have previously reported that the offspring of long-lived families, who have the propensity to reach an advanced age with limited clinical issues, seem to have higher levels (within normal range) of serum TSH and total 24-h secretion of TSH (67). Other studies have also shown that centenarians as well as their offspring had a higher level of TSH when compared to age matching controls (68, 69). Thus, these studies support the hypothesis that elevated TSH levels with ageing are not necessarily unfavorable.

### Other Sources of Within-Person Variation in TSH Levels

#### TPO-Ab Positivity

Evidence for the influence of several other factors on the within-person variation in TSH levels can be found in literature. For example, a positive association was found between thyroid peroxidase (TPO)-antibody positivity and TSH levels during early pregnancy in a large (*N*=11,212) individual participant-based meta-analysis (70). Similarly, a positive correlation between TPO-Ab positivity and TSH levels was observed, cross-sectionally as well as over months, within 21 individuals with subclinical hypothyroidism (60, 71).

#### BMI and Obesity

Furthermore, a positive correlation between TSH levels and body mass index (BMI) was found in a population-based (*N*=8,727) cross-sectional study (72). Similarly, median (IQR) TSH levels in obese (BMI ≥ 30 kg/m^2^) euthyroid subjects were somewhat higher [1.53 (1.07–2.23) *vs.* 1.47 (1.04–2.12) mU/L] than in the reference group (18.5–24.9 kg/m^2^) in a cross-sectional study with 11,224 participants (73). Moreover, TSH levels decreased significantly 12 months after weight loss due to bariatric surgery in 949 euthyroid patients with morbid obesity, which was more pronounced in the high-normal TSH group (74). BMI was positively associated with basal TSH secretion in healthy individuals, but not with pulsatile or total TSH secretion, and a higher BMI was associated with a delay in the nocturnal onset of the TSH surge in 117 healthy subjects with a mean age of 43 (range 22–77) years and a mean BMI of 26.8 (range 18.3–39.4) kg/m^2^ (23). In contrast, TSH levels were not different between a small group of overweight women (*N*=22) and women with normal weight (*N*=30) (75).

#### Selenium, Iodine, and Smoking

Although selenium deficiency is associated with impaired thyroid function, there is no evidence for a direct effect of selenium intake on TSH levels in individuals without severe selenium deficiency (76). Also, iodine intake can influence TSH levels; a positive correlation was found between urinary iodine concentrations and TSH levels in a large (*N*=6,565) Korean dataset (77). However, more research on the direct relationship between iodine intake and TSH levels is necessary. In the same Korean study, it was observed that mean serum TSH levels were lower in current smokers than in non-smokers, which was more apparent in iodine-deficient subjects (78). Other studies also showed that smoking is associated with lower TSH levels with modestly higher fT4 levels (79–81), which was reviewed before (82, 83).

#### Severe Illness

Critically ill patients exhibit a decrease in serum concentrations of thyroid hormones but with TSH levels in the normal range or slightly decreased, which is known as non-thyroidal illness syndrome (NTIS) or euthyroid sick syndrome (ESS). These effects of severe illness on thyroid function have been reviewed by others (84, 85). Another study found that pulsatile TSH secretion is diminished in 26 critically ill patients with a mean (SEM) age of 63 (2) years (86, 87).

#### Drugs and Xenobiotics

Many drugs can also affect thyroid function, including glucocorticoids, lithium, amiodarone, and antiepileptic drugs. A comprehensive review on the effects of drugs on TSH levels can be found elsewhere (88). Other xenobiotics, such as environmental endocrine disrupters, also have a large influence on thyroid parameters and metabolism (89).

## Potential Underlying Biological Mechanisms of Within-Person Variation in TSH Levels

### Potential Underlying Mechanisms of Pulsatile Secretion

The main sources of within-person variation in TSH levels have been discussed, but what are the biological mechanisms that underlie the sources of within-person variation in TSH levels? One of the main roles of the HPT axis is to respond to environmental changes in order to maintain homeostasis. TSH is a key player in this system, which is responsible for maintaining constant circulating levels of thyroid hormones within the normal ranges over time. TSH is highly responsive to different stressors, including inflammation (90) and environmental endocrine disrupters (89). To be able to respond quickly to changes in the environment, TSH is secreted in a pulsatile manner and the half-life of TSH is relatively short (17 to 93 min). It is not known whether T3 and T4 are secreted in a pulsatile manner from the thyroid gland. The half-lives of T3 and T4 are much longer (0.75 and 6.7 days respectively) than that of TSH (27). Free T3 exhibits a minor circadian rhythm and fT4 does not exhibit a circadian rhythm (27). However, it is not completely clear why TSH fluctuates in periods of minutes, hours, and months while fT4 levels stay relatively constant over time. Other pituitary hormones also have a short half-life and fluctuate over time—they are all designed to adapt in response to changes in the environment—while levels of peripheral hormones are more stable over time. For example, within the somatotropic axis, growth hormone (GH) is secreted in a pulsatile manner and fluctuates strongly over 24 h, while insulin-like growth factor 1 (IGF-1) is relatively stable over 24 h. It is known that manipulations of GH and IGF-1 have shared, but also distinct effects (91). Therefore, a hypothesis could be that TSH affects, besides similar, also different mechanisms and processes in the human body than thyroid hormones, which may explain the distinct fluctuations in levels over time between TSH and thyroid hormones. In line with this hypothesis it was observed, among others, that TSH receptors were located on T cells in the thymus and that TSH directly—independent of thyroid hormones—has an influence on the maturation of these T cells (92).

### Potential Underlying Mechanisms of Circadian Variation

The biological mechanisms that underlie the daily fluctuations in TSH levels are most likely related to the biological clock. The biological clock of the SCN drives the circadian rhythm of several processes, including hormones. The biological clock directly influences the circadian rhythm of TSH, but it was also found that the circadian rhythm of TSH is partly determined by the circadian rhythm of cortisol, which is completely dependent on the SCN (93). The circadian rhythms of TSH and cortisol are out of phase; TSH has a nocturnal surge in the early night while cortisol has its nadir around that time and its peak in the early morning. The circadian rhythms of cortisol and TSH being out-of-phase can be explained by the inhibitory influence of cortisol on serum TSH concentrations (94–98). Additionally, a strong negative cross-correlation between cortisol and TSH concentrations, with TSH following cortisol concentrations in the opposite direction after a time delay of 170 min, was observed in 38 healthy older individuals (99). Also, the biological clock influences processes of the sympathetic and parasympathetic nervous system. Differences in sympathetic/parasympathetic tone could possibly lead to alterations in end organ sensitivity, such as the thyroid gland. Although it is speculative, it could be suggested that the thyroid gland is less sensitive to TSH during the night and during winter seasons, and therefore higher TSH levels are required to keep fT4 levels constant. It is also known that other physiological processes exhibit a circadian rhythm, including metabolic processes. TSH plays an important role in metabolism and might therefore exhibit parallel fluctuations. TSH plays an important role in energy balance and distribution. Energy requirements also fluctuate over time, during the day as well as during seasons. Furthermore, it has been is hypothesized that TSH plays a role in maintenance and repair mechanisms, including bone turnover (100). These processes fluctuate over time, with a bone resorption marker, C-terminal cross-linked telopeptide of type 1 collagen (CTX-I), exhibiting a strong circadian rhythm (101).

### Potential Underlying Mechanisms of Seasonality: Environmental Temperature

A number of potential mechanisms may explain the circannual changes in TSH levels, with environmental temperature being the most apparent explanation which has been reviewed before (102). Two studies in healthy individuals and one study in patients with primary hypothyroidism under L-T4 treatment indeed observed a significant negative correlation between TSH levels and the environmental temperature (45, 48, 53). It is important to note that these studies were performed in Korea, China, and Japan where the environmental temperature is very low in winter. In contrast, two Italian studies did not find a significant correlation between TSH levels and environmental temperature (46, 55). It becomes less cold in winter in Italy than in Asia, which could explain the discrepancy in results. The seasonal fluctuation might therefore be more pronounced in particularly cold climates. Most studies on circannual changes in TSH levels were performed in the United States, Asia, and Europe. However, some studies were performed in Antarctica and at the South Pole, where the effect of long-term cold exposure could be studied in personnel of the McMurdo and South Pole research stations. Mean TSH levels increased by approximately 30% during a 9-month Antarctic residence in nine healthy young subjects (103) and the TSH response to TRH increased by approximately 50% in 17 healthy young men (104). Similarly, mean TSH levels increased in ten healthy men after a 54-weeks Antarctic expedition (105) and comparable results were obtained in a larger study of 187 workers at the McMurdo and South Pole research stations (106). These changes in thyroid function are known as the polar T3 syndrome, which is not only characterized by increased TSH levels, but also by alterations in T3 kinetic parameters such as an increase in serum clearance of T3, possibly explained by increased tissue uptake (107). This thyroidal response to cold might be required to keep the core body temperature constant. Further support for the effect of environmental temperature on TSH levels are observations in bears. In a study with six American black bears it was found that the TSH response to TRH administration was prolonged, delayed, and exaggerated during hibernation in winter while (f)T3 and (f)T4 levels were decreased (108). In addition, T3 levels increased stronger after TRH administration during hibernation than after hibernation.

### Potential Underlying Mechanisms of Seasonality: Length of Day

Studies in animals indicated that length of day, and possibly melatonin (109), plays a role in the seasonal variation in TSH levels, which has been reviewed by others (110). Animals that breed seasonally exhibit seasonal variations in numerous processes and functions, including reproduction, fattening, hibernation, and migration, which are regulated *via* photoperiod-regulated systems. It was for example found that the simulation of a long-day in the quail induced the secretion of TSH from the pars tuberalis (PT) of the anterior pituitary gland (111). Moreover, light induced the expression of the gene encoding type 2-iodothyronine deiodinase (Dio2), which converts T4 to T3, in quails (112). The underlying mechanism is possibly that TSH and/or thyroid hormones stimulate the secretion of gonadotropin-releasing hormone and gonadotropin leading to gonadal growth, which is important for reproduction (111, 113). Humans however are not seasonal breeders, so these mechanisms could be of greater importance in animals than in humans. Nevertheless, understanding the seasonal mechanisms in animals might lead to better understanding of seasonality in humans. The thyroid also plays a role in other transitions to different physiological states that exist in nature, for example in the metamorphosis of the tadpole to a frog (114–116).

### Potential Underlying Mechanisms of Age-Related Changes

Several hypotheses exist why there is an increase in population TSH levels with increasing age (117, 118). At a population level, the higher median TSH levels in older individuals compared to younger individuals might be explained by 1) age-dependent selective survival of individuals with elevated TSH levels or by 2) a birth cohort effect since different birth cohorts have been exposed to different environmental factors which could have influenced their TSH levels. At the organism level, age-related increase in TSH levels might be caused by 1) decreased TSH bioactivity, 2) diminished response of the thyroid gland to TSH stimulation, or 3) diminished sensitivity of the pituitary and/or hypothalamus to the negative feedback of thyroid hormones. The last hypothesis however would be associated with an increase in thyroid hormone levels, which is not supported by literature. The lack of evidence for increased thyroid hormone levels in response to diminished sensitivity of the pituitary and/or hypothalamus could be explained by enhanced thyroid hormone turnover in peripheral tissues or greater clearance of thyroid hormones from the circulation (67).

## Clinical Implications Of Within-Person Variation in TSH Levels

TSH levels fluctuate over time within a person, both in euthyroid individuals and in most patients with thyroid disorders. If clinicians are not aware of fluctuations in TSH levels within an individual patient, this could lead to under- or overdiagnosis, and to inadequate prescription of thyroid medication to patients with thyroid disease. Since the mean TSH levels and the prevalence of subclinical hypothyroidism is increasing with age, this is especially important for the diagnosis and follow-up of older patients. Also, for clinical scientific research is it important to consider within-person variations in TSH levels over time, since this could cause more heterogeneity in research outcomes.

### Season of Testing and the Transition of Subclinical Hypothyroidism to Euthyroid

Whether the sampling time has an influence on the assignment of an individual to a clinical category of thyroid status is not known. In contrast, it was shown that the season of thyroid testing played a role in the transition of patients between subclinical hypothyroidism and euthyroid status (48). In a large (*N*=29,847) retrospective longitudinal study, 57.9% of subclinical hypothyroid individuals (*N*=1,751) reverted to euthyroid TSH levels during a median follow-up of 36 months. Normalization of subclinical hypothyroidism to euthyroidism was increased during summer-fall follow-up. The opposite was true as well; the transition from euthyroidism to subclinical hypothyroidism was more likely in individuals who had their follow-up TSH measurement in winter or spring (48). Also, other studies showed that TSH levels of patients with subclinical hypothyroidism can normalize to euthyroid levels without intervention. In total, 40% of the participants (*N*=2,936) aged above 65 years from a longitudinal follow-up study, the Birmingham Elderly Thyroid Study, who classified as being subclinical hypothyroid at baseline normalized to euthyroid status without intervention over a period of 5 years (119). However, the authors also concluded that TSH concentrations were quite stable over time; 61% of the participants had a repeated TSH concentration within 0.5 mU/L of their original result. A small study (*N*=107) found that 37.4% of older (above 55 years) patients with subclinical hypothyroidism showed normalization of their TSH levels during a mean (range) follow-up of 31.7 (6–72) months (120, 121). Whether the normalization was related to the season in which follow-up measurements were taken was not reported in these two studies.

### Clinical Reference Range

Studying the within- and between-person variations in TSH levels revealed that the within-person variation is smaller than the between-person variation (3, 60, 122). A seemingly small change in TSH level within the population reference interval might therefore be large—and potentially even pathological—for an individual. Indeed, a meta-analysis showed that variations in TSH levels within the population reference range are associated with adverse health outcomes, including cardiovascular risk factors, metabolic parameters, and increased risk of osteoporosis and fracture (123). It has therefore been recommended to determine individual trends and variations in experimental clinical research, and thereby even establish one’s individual TSH reference range, by performing repeated measurements (124). Although this strategy is not feasible in routine clinical practice, it might be worthwhile to explore the possibility to monitor patients who are diagnosed with subclinical hypothyroidism more regularly since their TSH level is often close to the upper reference limit. Procedures for regular monitoring of a subclinical hypothyroid patient’s TSH levels have been recommended by others (60). Moreover, Hoermann et al. stated that there should be more attention in clinical practice for the interrelationships between TSH and thyroid hormones (125). Mathematical models have shown that the relationship between TSH and fT4 is not log-linear as previously thought, but much more complex, dynamic, and individual (125).

## Conclusions

**Figure 3:**
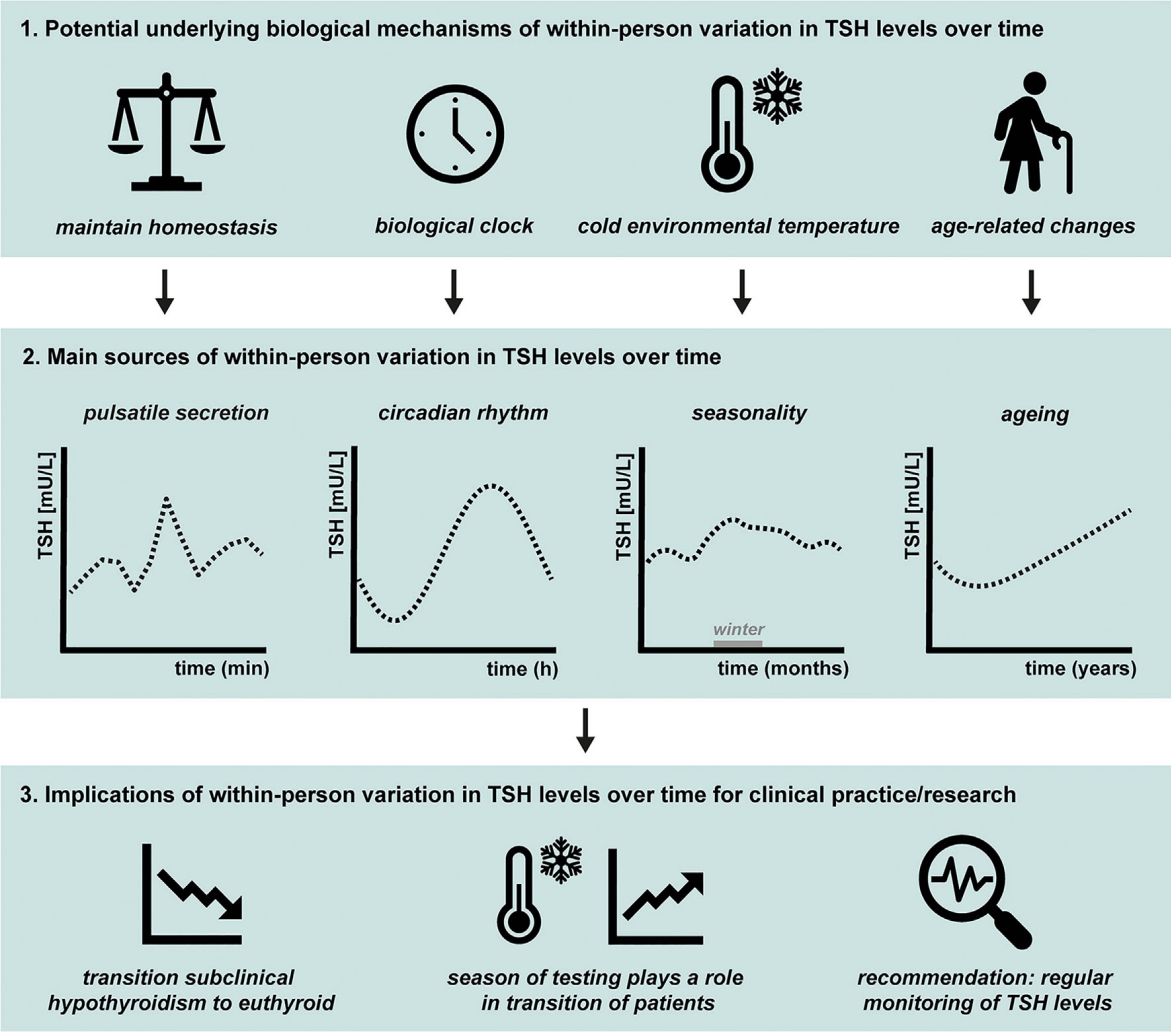
Graphical summary of 1) the potential underlying biological mechanisms of within-person variation in thyroid-stimulating hormone (TSH) levels over time, 2) the main sources, and 3) the clinical implications. The main topics discussed in this review are illustrated. The main sources of within-person variation in TSH levels over time are pulsatile secretion, circadian rhythm, seasonality, and ageing, as illustrated in 3.2. The main hypotheses for the underlying mechanisms of within-person variations are illustrated in 3.1. The pituitary needs to respond quickly to changes in the environment to maintain homeostasis, which is one of the potential underlying biological mechanisms of pulsatile secretion of TSH. The biological clock regulates the circadian rhythm of many physiological processes including pituitary hormones and energy metabolism. The seasonality in TSH levels are possibly caused by changes in environmental temperature, with studies indicating generally highest levels during winter season. Multiple studies showed that on a population level, TSH levels generally increase with age. However, the size and rate of the increase within an individual is not completely clear. Finally, the implications of within-person variation in TSH levels over time for clinical practice and scientific research are illustrated in 3.3. Within-person variation in TSH levels is not only present in healthy euthyroid individuals, but also seen in patients with (un)treated thyroid disease. It occurs frequently that subclinical hypothyroid patients normalize to euthyroid levels over time without intervention and the season of thyroid testing plays a role in the transition of patients between subclinical hypothyroidism and the euthyroid status. We recommend regular monitoring of an individual’s TSH levels by obtaining repeated measurements so different sources of within-person variations can be considered.

A graphical summary of this review is provided in **Figure 3**. TSH levels fluctuate over time within a person, in euthyroid individuals as well as in most (un)treated patients with thyroid disorders, ranging from minutes, hours, months, to years. These within-person variations in TSH levels are mainly caused by pulsatile secretion, circadian rhythm, seasons, and ageing. If TSH is fluctuating around the upper reference range limit, these marginal changes could lead to diagnosis of subclinical hypothyroidism, especially in older adults. However, elevated TSH levels can return to euthyroid levels in a short period of time without intervention, which can be explained by the different sources of within-person variation. Therefore, we recommend considering the fluctuations in TSH levels and their potential underlying biological mechanisms in clinical practice and when performing clinical research. For example, by adaptation of the TSH reference limits, specifically the upper TSH reference limit, for the seasonal variations, circadian rhythm, and age. Future studies should aim to find approaches to provide a more personalized reference range for thyroid disease diagnosis and clinical treatment which considers the different sources of within-person variation in TSH levels. Moreover, we propose that there should be more attention in clinical practice for the relationship between TSH and fT4 levels, for example using a TSH-fT4 nomogram. Ideally, an algorithm will be developed which includes the individual TSH-fT4 nomogram together with the time of day and season of the blood sampling, the age of the patient, and possibly other factors that influence TSH levels, such as pre-analytical and analytical variation.

## Author Contributions

All discussed the content, main message, and structure of the review. EV conducted literature search and wrote the first draft. FR and DV reviewed the text and edited some parts. All discussed the content of the figures. FR and EV made the figures. All authors contributed to the article and approved the submitted version.

## Funding

This work was supported by the European Commission project THYRAGE (Horizon 2020 research and innovation programme, 666869).

## Conflict of Interest

The authors declare that the research was conducted in the absence of any commercial or financial relationships that could be construed as a potential conflict of interest.
